# The Deadly Details: How Clear and Complete Are Publicly Available Sources of Human Rabies Information?

**DOI:** 10.3390/tropicalmed10010016

**Published:** 2025-01-07

**Authors:** Natalie Patane, Owen Eades, Jennifer Morris, Olivia Mac, Kirsten McCaffery, Sarah L. McGuinness

**Affiliations:** 1 Infectious Diseases Epidemiology Unit, School of Public Health and Preventive Medicine, Monash University, Melbourne, VIC 3004, Australia; npat0036@student.monash.edu (N.P.); owen.eades@monash.edu (O.E.); 2 Independent Consumer Advisor, Melbourne, VIC 3000, Australia; 3 Sydney Health Literacy Lab, School of Public Health, University of Sydney, Sydney, NSW 2006, Australia; olivia.mac@sydney.edu.au (O.M.); kirsten.mccaffery@sydney.edu.au (K.M.); 4 Department of Infectious Diseases, Alfred Hospital, Melbourne, VIC 3004, Australia

**Keywords:** rabies, prevention, vaccination, readability, health literacy, public health, patient information

## Abstract

Human rabies is preventable but almost always fatal once symptoms appear, causing 59,000 global deaths each year. Limited awareness and inconsistent access to post-exposure prophylaxis hinder prevention efforts. To identify gaps and opportunities for improvement in online rabies information, we assessed the readability, understandability, actionability, and completeness of online public rabies resources from government and health agencies in Australia and similar countries, with the aim of identifying gaps and opportunities for improvement. We identified materials via Google and public health agency websites, assessing readability using the Simple Measure of Gobbledygook (SMOG) index and understandability and actionability with the Patient Education Materials Tool for Print materials (PEMAT-P). Completeness was assessed using a framework focused on general and vaccine-specific rabies information. An analysis of 22 resources found a median readability of grade 13 (range: 10–15), with a mean understandability of 66% and mean actionability of 60%; both below recommended thresholds. Mean completeness was 79% for general rabies information and 36% for vaccine-specific information. Visual aids were under-utilised, and critical vaccine-specific information was often lacking. These findings highlight significant barriers in rabies information for the public, with most resources requiring a high literacy level and lacking adequate understandability and actionability. Improving readability, adding visual aids, and enhancing vaccine-related content could improve accessibility and support wider prevention efforts.

## 1. Introduction

Rabies is a viral disease spread through the saliva of infected mammals [1]. It poses a serious public health challenge in over 150 countries and territories, particularly in Asia and Africa. Transmission occurs via bites, scratches, and licks to broken skin from a range of domestic and wild mammals [1,2]. Dog-mediated rabies accounts for over 99% of human cases [1,3]. Once clinical symptoms appear, rabies is almost invariably fatal [4]. However, timely post-exposure prophylaxis (PEP), involving wound care, multiple doses of rabies vaccine, and, for those previously unvaccinated, rabies immune globulin (RIG), effectively prevents the disease [1,5,6]. Despite its safety and effectiveness, global disparities in access to rabies vaccine and RIG pose barriers for those in need [7,8,9]. Pre-exposure prophylaxis (PrEP), achieved through 2–3 doses of the rabies vaccine, primes the immune system, leading to an anamnestic response once a post-exposure rabies vaccine is administered [5,10,11]. It simplifies PEP by reducing the required number of vaccine doses, and eliminating the need for RIG in the event of subsequent exposure [7].

Rabies causes an estimated 59,000 human deaths annually worldwide. Cases of human rabies imported into non-endemic areas are comparatively rare, averaging 3.5 imported cases per year [12]. However, due to their deadly nature, they frequently prompt public health alerts and media attention. A significantly higher number of travellers have potential rabies exposures, with an estimated incidence rate of 4 per 1000 travellers per month [13]. Unfortunately, many of these travellers do not seek or receive complete PEP after potential exposures [12,14]. Access to RIG in particular is scarce, with only 5–20% of travellers receiving RIG in the country of exposure when indicated [12]. This is underpinned by logistical factors involving RIG availability, cost, and storage, as well as lack of awareness about when RIG is indicated [12].

The WHO recommends that travellers to rabies-endemic areas undergo an individual pre-travel risk assessment. This assessment should consider the remoteness and rabies epidemiology of the destination, as well as the duration of stay in rabies-endemic areas [15]. Travellers intending to stay in remote rural areas with limited access to PEP, or those engaging in extensive outdoor activities that may increase their proximity to animals, should consider receiving PrEP before travelling [15]. Even short-term travellers may be at risk of rabies, and PrEP may be considered based upon their intended activities and potential exposure risks [16,17]. Receiving PrEP before travel reduces the risk associated with limited PEP access abroad and helps conserve resources for endemic populations [1]. Despite its benefits, the uptake of rabies PrEP remains limited due to factors such as limited awareness of rabies severity and preventive options, concerns about vaccine costs, and the logistical challenges of completing a vaccine schedule that requires 2–3 doses spaced over time [18,19].

Accessible health information is vital for empowering individuals to make informed decisions about their health, particularly in contexts involving disease prevention and management. In recent decades, there has been a notable shift towards using online health information as a preferred source globally [20]. However, despite its potential to influence health-related decisions [20] and promote healthier behaviours [21], studies indicate online rabies information does not effectively facilitate pre-travel vaccination uptake among travellers to high-risk areas [22,23]. This highlights a gap between existing resources and user needs. Health literacy is crucial for navigating online health information [24], yet readability, understandability, and actionability often fail to meet diverse health literacy needs [25,26,27,28,29,30]. This disparity risks widening health inequities and further marginalising disadvantaged populations.

Our study aimed to assess the readability, understandability, actionability, and completeness of public-facing rabies information from various government and peak public health agency websites to identify gaps and opportunities for improvement. We hypothesised these resources exceed the recommended eighth-grade reading level, fall short of adequate thresholds for understandability and actionability, and do not fully address the information needs of the public.

## 2. Materials and Methods

We evaluated publicly available online rabies information from government and peak public health agencies in Australia and other English-speaking countries that are free of canine (dog) rabies (Supplementary S1). We focused on officially badged sites intended for public use. We excluded commercial sites, resources addressing lyssaviruses other than rabies, and content designed specifically for healthcare providers or veterinarians.

### 2.1. Search Strategy

Using Google, we conducted a keyword search to identify publicly available rabies information on government and public health websites from Australia, Canada, New Zealand, the United States of America, and the United Kingdom in March and April 2024. (Supplementary S2, Figure S1). We supplemented this with direct searches on key websites, including relevant departments of health, the World Health Organization (WHO), and the Centers for Disease Control and Prevention (CDC).

### 2.2. Assessments

Assessments of readability, understandability, actionability, and completeness were conducted between 3 March and 2 May 2024. An updated version of the CDC website, which emerged after this analysis period, was analysed separately.

Readability is a measure of text complexity, operationalised by several mathematical formulas that use specific text features to produce a readability score, typically expressed as a grade level corresponding to U.S. school grades [31]. For this assessment, we used the Sydney Health Lab Literacy (SHeLL) Health Literacy Editor [32], an online tool that applies the Simple Measure of Gobbledygook (SMOG) index. The SMOG index estimates the reading level based on the count of polysyllabic words and sentence length, with a grade eight level considered acceptable for general audiences [32,33,34]. We followed a text preparation protocol adapted from the Health Literacy Editor guidelines (Supplementary S3) [32]. For resources with linked pages, each linked page was assessed separately, and a reading grade score was generated from the median score of all linked pages.

We evaluated understandability and actionability using the validated 26-item Patient Education Materials Assessment Tool for printable materials (PEMAT-P) (Supplementary S4), which addresses the limitations of relying solely on readability in gauging accessibility [35]. PEMAT-P measures how well readers can process and explain key points in the material (understandability, items 1–19) and how well readers can identify actionable steps from the information presented (actionability, items 20–26) [35]. Each item is rated as 0 (disagree), 1 (agree), or N/A (not applicable). Two researchers (OE and NP) independently rated each resource, resolving discrepancies through discussion. Scores reflect the proportion of ‘agree’ responses across the items in each domain, with scores ≥ 70% indicating adequate understandability or actionability [35]. Resources with linked pages were assessed collectively.

Completeness was assessed using a 23-item framework designed by study investigators (content experts and a consumer representative) based upon previous research [36] (Supplementary S5). The framework included two domains: general rabies information (items 1–14) and vaccine-specific information (pre- and post-exposure; items 15–23). Items were scored 2 (complete), 1 (partial), or 0 (not provided). Two researchers (OE and NP) independently rated each resource, resolving discrepancies through discussion. The completeness score represents the proportion of items rated as ‘partial’ or ’complete‘ (score ≥ 1) for each resource. The resources with linked pages were assessed collectively.

### 2.3. Data Analysis

Data were recorded and analysed using Microsoft Excel and Stata (version 18). We calculated the median and interquartile range (IQR) for readability, along with ranges for resources with linked pages. We calculated mean understandability and actionability scores, and identified the items with the highest proportion of ‘disagree’ scores across all resources. Similarly, we calculated mean completeness for both domains and identified the items with the highest proportion of ‘information not provided’ scores. When raters differed, discrepancies were handled by selecting the lower score. Inter-rater agreement for understandability, actionability, and completeness was calculated prior to any adjustments, using kappa statistics [37].

## 3. Results

We assessed 22 sources of online rabies information from 15 government and public health agency websites. Table 1 summarises key findings, including individual scores for readability understandability, actionability, and completeness, along with the median readability level and mean understandability, actionability, and completeness scores. The median word count and number of pages is also presented. Inter-rater agreement based on initial scoring (before discussion and adjustments) was deemed substantial (Cohen K > 0.61).

Most resources were single-page websites, with an overall median word count of 798 words. Resources with more text typically scored higher for completeness but had worse readability scores.

Median overall readability was grade 13 (IQR 11–14; range 10–15), with no resource meeting the ideal grade eight reading level for general audiences. Mean understandability was 66% (SD = 13%; range 42–87%), with 10 resources (46%) achieving the recommended 70% threshold. Mean actionability was 60% overall (12%; 33–83%), with only four resources (18%) meeting the 70% threshold.

**Figure 1:**
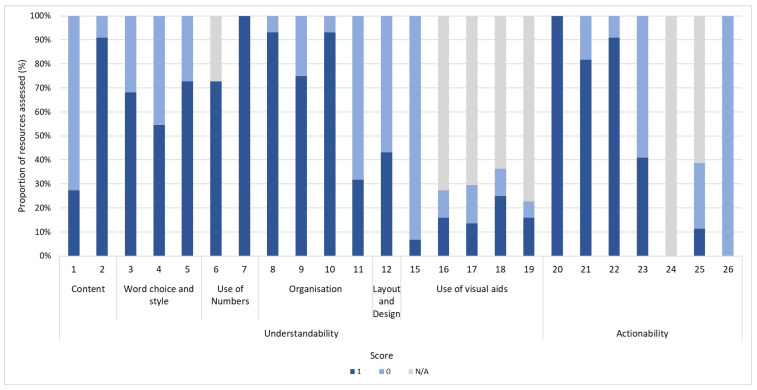
**Distribution of PEMAT-P scores by item across all resources.** Items 1–19 of PEMAT-P assess understandability, while items 20–26 assess actionability. Each bar in columns indicates proportion of resources where raters either agreed (1) or disagreed (0) that material met specific criterion, or deemed it N/A (not applicable) for that specific item.

Figure 1 illustrates the distribution of PEMAT-P scores by item across resources. While all resources identified at least one actionable step (item 20), no resource made meaningful use of visual aids to prompt user action (item 26) and only 9% (2/22) did so to make content more easily understood (item 15).

Mean completeness for general rabies information was 79% (13%; 50–100%), and for vaccine-specific information it was 36% (30%; 0–100%). A completeness score of ≥70% was achieved by 17 resources (77%) for general rabies information, and three resources (14%) for vaccine-specific information.

**Figure 2:**
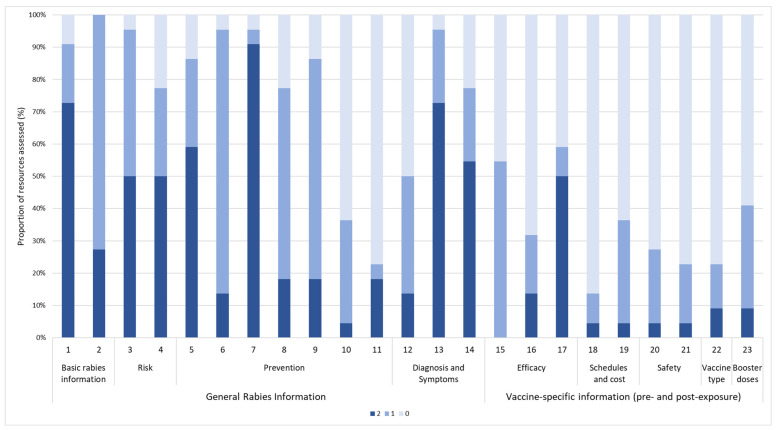
**Distribution of completeness scores by item across all resources.** Items 1–14 assess general rabies information, while items 15–23 assess information specific to rabies vaccination (both pre- and post-exposure). Each bar in columns represents proportion of resources rates scored as providing complete (2) or partial information (1), or did not provide any information (0) for that specific item.

Figure 2 shows the distribution of completeness scores by item across all resources. For general rabies information, high proportions of ‘information not provided’ ratings were observed for item 11 (risk of disease following exposure; 77% scoring zero), item 10 (availability of treatment; 64%), and item 12 (diagnosis; 50%). For vaccine-specific information, items with high proportions of ‘information not provided’ ratings were item 18 (availability of different vaccine schedules; 86% scoring zero), item 21 (vaccine safety based on individual factors; 77%), item 22 (type of vaccine; 77%), item 16 (duration of protection; 73%), and item 20 (vaccine adverse events; 73%).

After the study period, the CDC rabies website was updated. We reassessed the new version using the same criteria. Compared to the previous version, median readability decreased from grade 14 (range: 9.9–17.8) to grade 13 (9.5–15.4). Mean understandability improved from 53% to 78%, while actionability remained at 83%. The proportion of items with partial or complete information decreased from 100% to 89% for general rabies information, and from 100% to 17% for vaccine-specific information.

## 4. Discussion

To our knowledge, this study is the first to systematically evaluate publicly available rabies information using validated measures of health information quality. Our findings highlight significant shortcomings in readability, actionability, understandability, and completeness of current resources for individuals with varying levels of health literacy.

None of the resources analysed met acceptable reading levels for general audiences (grade eight). Previous research from Australia and globally has shown that online public health information often exceeds recommended readability levels [26,28]. In particular, vaccine information often demands higher health literacy than other public health topics, such as mask use or physical distancing [21,23]. Poor readability can impede self-management and decision-making, particularly for individuals with lower health literacy levels [26].

Our study also revealed a lack of actionable content in online rabies information, largely due to the inadequate use of visual aids and practical tools that support user action and decision-making. This aligns with findings from research on online COVID-19 resources and immunisation materials for migrants and refugees, which similarly highlighted deficiencies in actionability and a scarcity of tangible tools or visual aids [27,38]. The under-utilisation of visual aids is concerning, as they can greatly enhance understanding and decision-making, especially for individuals with low health literacy [29,38,39,40]. Effective visual aids, such as icon arrays, maps, and graphics, could enhance comprehension of rabies risk, vaccine efficacy, safety, and key prevention actions, such as wound washing and seeking medical assistance if exposed.

Due to the lack of validated tools for assessing information completeness in public-facing health resources, we created our own framework based on previous research [36]. Our completeness assessment revealed significant gaps in rabies information across many resources, particularly in vaccine-specific details. These deficiencies may contribute to the low uptake of pre-travel rabies vaccination among travellers to endemic areas, as noted in previous studies [22,23,41,42]. Only three resources (from the CDC, NaTHNaC, and NHS) scored ≥70% in both general and vaccine-specific information. While the CDC and NaTHNaC resources were comprehensive, their lengthy content and university-equivalent (grade 14) reading levels limited accessibility. Recent updates to the CDC website improved understandability but reduced completeness, emphasising the need for a balanced approach that ensures health resources are both thorough and accessible, empowering members of the public to make well informed health decisions.

Decision-making around rabies pre-exposure vaccination (PrEP) is complex and influenced by individual, travel, and logistical factors [43]. International opinions on which travellers should receive PrEP and the preferred schedules vary significantly [44]. However, most guidance suggests that healthcare professionals conduct risk assessments for travellers to rabies-endemic areas, considering factors such as the likelihood of animal interaction and access to rabies PEP and emergency medical care. Children are often advised to receive PrEP due to their smaller stature, which increases the risk of exposures in higher-risk areas like the head and neck, as well as their tendency to interact with animals, and potential inability to report minor exposures [43,45]. PrEP is also often recommended for those undertaking longer trips, engaging in outdoor activities, or who will be more than 48 h away from facilities providing appropriate PEP [15,43]. Given the limited access to PEP in many popular travel destinations and the frequency of incomplete PEP [8,12], PrEP may be advisable for significantly more travellers than those who currently receive it. Ensuring that prospective travellers have access to clear and complete information enables them to make informed decisions about their health and safety whilst abroad.

Barriers to rabies PrEP include the multi-dose schedule, out-of-pocket costs, and lack of awareness [43,46,47,48,49]. Multiple studies have highlighted cost as a major barrier [47,48,49]. The cost of PrEP may be reduced by decreasing the number of doses or administering smaller doses via the intradermal (ID) route [43,50,51]. Recent WHO guidelines now recommend a two-dose PrEP schedule instead of the previous three-dose regimen, but logistical and financial barriers persist [15,46]. Some studies suggest that a single dose of an intramuscular (IM) rabies vaccine can effectively prime the immune system and could potentially replace the current standard two-dose regimen [52]. However, the efficacy of a single-dose schedule continues to be debated [46,53,54] with current WHO guidelines still recommending a complete PEP course with RIG in the event of exposure after receiving a single pre-exposure dose [15]. Immunity after a complete rabies pre-exposure vaccination schedule is long-lasting and boostable over long time intervals [52], making rabies vaccination a valuable investment for travellers who frequently visit rabies-endemic areas.

Education strategies are essential for communicating rabies risk to travellers and encouraging timely pre-travel health consultations [55]. Improved communication between travellers and clinicians can increase PrEP uptake and influence travellers to select lower risk destinations and activities [55]. However, recent studies indicate that current pre-travel rabies education falls short of meeting travellers’ needs [56]. While online health information has the potential to support informed decision-making about PrEP, its actual impact on PrEP uptake is currently limited [22,23]. The literature emphasises the need for better traveller education on rabies risks, vaccine availability, and risk-reducing behaviours, particularly for last minute travellers [43,45,47,56,57,58].

Efforts to address these issues include tools like Croughs and Soentjens’ risk scoring system to identify travellers eligible for PrEP [44] and the CDC’s country classification system to guide healthcare providers and policymakers [8]. While some publicly accessible websites (e.g., CDC, the WHO) offer country-specific rabies status information (Supplementary S6), these resources are not specifically designed for the general public, and do not address other barriers to rabies vaccination, such as low risk perception [23,59].

To effectively tackle these challenges, there is a need for well-designed, public-facing online resources. Public health websites should be enhanced with clear, comprehensive information, utilising visual aids and accessible language. Given the complexities of rabies vaccine decision-making, developing targeted decision aids could significantly improve communication between travellers and healthcare providers and support travellers to make informed choices [60,61]. We advocate for further research into the factors influencing rabies PrEP uptake, as well as the development and evaluation of decision aids to support informed decision-making for rabies vaccination.

Finally, it is crucial to consider the implications of rabies PrEP uptake on global vaccine equity. The current global burden of rabies is highly inequitable, with daily fatalities from dog-mediated rabies in low- and middle-income countries (LMICs) far exceeding the number of deaths caused by dog-mediated rabies in high-income countries this century [62]. In resource-limited settings, rabies biologics, particularly RIG, are scarce and highly valued [63]. A full PEP course, involving multiple doses of a rabies vaccine and RIG, is costly to produce, and global supply is unevenly distributed, with limited availability in LMICs where populations are most at risk [63,64,65]. The lack of RIG, particularly in rural areas of LMICs, often prevents patients in these areas from accessing life-saving treatment [63]. This scarcity raises equity concerns, as travellers may use resources that are desperately needed by local residents [64].

Dog vaccination is an effective method of combating rabies and was crucial in eliminating rabies in high-income countries. However, dog vaccination efforts remain limited in LMICs [62]. While the WHO recommends PrEP for populations in highly endemic areas with limited access to PEP [15], its availability and uptake remain low, hindered by economic and logistical challenges [64]. Travellers to rabies-endemic areas arguably have an ethical responsibility to consider rabies PrEP, not only to protect themselves, but also to potentially conserve RIG for local populations and alleviate the burden on healthcare systems in LMICs. Providing travellers with complete, understandable information, such as an online decision aid, may improve PrEP uptake and help conserve critical resources. Additionally, rabies PrEP reduces the risk of travellers facing challenges in accessing life-saving PEP while abroad or needing to alter travel plans to obtain appropriate PEP [43].

### 4.1. Strengths, Limitations, and Generalisability

Our study used objective and validated tools (SMOG index and PEMAT-P) to evaluate readability, understandability, and actionability. However, it is important to acknowledge the inherent limitations of these tools. Many traditional readability formulas, while useful, can oversimplify comprehension by focusing solely on text features like word length and sentence structure. We chose to use the SMOG index due to its ease of use and suitability for healthcare applications, as it tends to provide consistent results and has a lower likelihood of underestimating reading levels compared to other formulas [66]. Additionally, we used the SHeLL Health Literacy editor, which was shown to provide more accurate assessments than other online readability calculators [67].

To address some limitations associated with relying solely on readability, we combined these assessments with evaluations of understandability, actionability, and completeness. While PEMAT is validated by both healthcare professionals and lay people, it can be subject to individual interpretation. To mitigate this, we employed two independent evaluators and an adjudication process for discrepancies, achieving good inter-rater agreement before adjudication. To assess the completeness of rabies information, we used a purpose-built framework, developed with content experts, a consumer representative, and prior research. This approach adds a unique strength that distinguishes our work from other health literacy studies.

While our analysis focused on English-language sources from authorised websites in countries with rabies profiles similar to Australia, this scope may not fully represent the information available in countries where rabies is endemic or present in companion animals. Additionally, we did not examine commercial sources, such as travel clinic websites, which the public might also use, further limiting the generalisability of our findings.

### 4.2. Implications

Our study adds valuable insights into the broader body of evidence concerning publicly available sources of health information and health literacy, which may inform best practice guidelines for online health information. These findings will directly inform our development of a rabies vaccine decision aid. We also plan to report findings to the administrators of the analysed resources, potentially prompting updates and improvements.

## 5. Conclusions

Despite the increased reliance on online health information, our findings reveal significant shortcomings in the readability, actionability, and understandability of online rabies information for the public. Most sources demanded health literacy level above the recommended grade eight for general audiences and fell below acceptable thresholds for understandability and actionability (70%). Critical information was frequently missing, and visual aids were under-utilised. Improving online rabies information is essential for equitable access to life-saving information. Enhancing readability, completeness, understandability, and actionability through visual aids and tangible tools will better equip the public to make informed health decisions.

## Figures and Tables

**Table 1:** Readability, understandability, actionability, and completeness scores and number of words and pages of each resource.

Country/Jurisdiction	Governing Body	Page Name	Readability SMOG Index Grade Reading Level Median (Range ^a^)	PEMAT-P Score Proportion of Items Rated ‘Agree’ (Standard Deviation)		Completeness Proportion of Items Scored ≥ 1 (Standard Deviation)		Number of Pages	Number of Words ^b^
				Understandability	Actionability	General Rabies Information	Vaccine-Specific Information		
Australia	Australian Federal Government	*Rabies*	10	79%	67%	86%	44%	1	1861
ACT	ACT Government	*Rabies and Australian Bat Lyssavirus*	13	42%	60%	79%	22%	1	669
NSW	NSW Government	*Rabies and Australian bat lyssavirus infection fact sheet*	14	62%	60%	86%	44%	1	1335
		*Rabies information for travellers*	15	75%	70%	79%	11%	1	733
NT	NT Government	*Australian bat lyssavirus and rabies*	11	62%	60%	50%	0%	1	392
QLD	QLD Government	*Rabies*	13	54%	50%	86%	22%	1	1125
SA	SA Government	*Rabies virus and Australian bat lyssavirus*	13	77%	60%	86%	22%	1	830
		*Rabies vaccines*	13	69%	60%	57%	56%	1	510
VIC	Victorian State Government	*Rabies*	13	63%	50%	79%	33%	1	762
WA	Western Australia Government	*Rabies and lyssavirus*	12	69%	60%	79%	33%	1	765
USA	U.S. Centers for Disease Control and Prevention	*Rabies*	11	73%	80%	79%	22%	1	742
		*Rabies* ^c^	14 (9.9–17.8)	53%	83%	100%	100%	21	10,476
Canada	Government of Canada	*Rabies*	12	77%	40%	79%	0%	1	721
		*Rabies: Symptoms and Treatment* ^c^	11 (10.4–12.6)	79%	67%	93%	0%	4	2110
UK	NaTHNaC	*Rabies*	14	43%	50%	93%	100%	1	3909
	Public Health Scotland	*Rabies*	11	69%	67%	86%	56%	1	993
	NHS	*Rabies* ^c^	10 (9.3–9.7)	77%	60%	79%	100%	2	1686
	Public Health England	*Rabies information for travellers*	13	75%	80%	57%	0%	1	665
		*Rabies information for travellers (leaflet)*	12	87%	60%	64%	22%	1	647
WHO	WHO	*Rabies*	15	47%	33%	86%	22%	1	1500
		*Overview Rabies* ^c^	14 (13–14.4)	44%	60%	50%	22%	3	743
		*Frequently asked questions about rabies for the general public*	14	75%	50%	93%	33%	1	3491
**OVERALL**			**13 (10–15)**	**66% (13%)**	**60% (12%)**	**79% (13%)**	**36% (30%)**	**1 ^d^**	**798 ^d^**

^a^ For resources with multiple linked pages, grade reading level was evaluated for each individual page and is presented as median (range) across all pages. ^b^ Obtained from SHeLL editor. Does not include list of headings/table of contents, contact information, references/sources, footnotes, or any information directed towards health professionals. ^c^ Landing page for a series of linked pages. ^d^ Median across all resources.

## Data Availability

The original contributions presented in this study are included in the Supplementary Materials.

## Supplementary Materials

The following supporting information can be downloaded at: https://www.mdpi.com/article/10.3390/tropicalmed10010016/s1. Supplementary S1: Website URLs; Supplementary S2: Search strategy; Supplementary S3: Readability protocol; Supplementary S4: PEMAT-P framework; Supplementary S5: Completeness framework. The raw data are contained in the following link: tmid_supplementary-raw-data_2024-11-13. Supplementary S6: Country-specific rabies status information.
