# Supplementary material for: The Deadly Details: How Clear and Complete Are Publicly Available Sources of Human Rabies Information?
Source: Trop Med Infect Dis. 2025 Jan 7;10(1):16. doi: 10.3390/tropicalmed10010016 (PMC11768519; doi:10.3390/tropicalmed10010016)
Supplement: Supplementary file 1 [file tropicalmed-10-00016-s001.zip › tropicalmed-3329770-supplementary.pdf]

Supplementary Materials

Article Title: The deadly details: how clear and complete are publicly available sources of rabies information?

Supplementary S1 – Website URLs

Table S1. List of websites evaluated per country/jurisdiction

**Country/Jurisdiction and site name**	**URL**
**AUSTRALIA**	
Rabies	https://www.healthdirect.gov.au/rabies
**Australian Capital Territory**	
Rabies and Australian Bat Lyssavirus	https://www.act.gov.au/health/topics/diseases/rabies-and-australian-bat-lyssavirus
**New South Wales**	
Rabies and Australian bat lyssavirus infection fact sheet	https://www.health.nsw.gov.au/Infectious/factsheets/Pages/rabies-australian-bat-lyssavirus-infection.aspx
Rabies information for travellers	https://www.health.nsw.gov.au/Infectious/factsheets/Pages/rabies-travel.aspx
**Northern Territory**	
Australian bat lyssavirus and rabies	https://nt.gov.au/wellbeing/health-conditions-treatments/viral/bat-lyssavirus-rabies
**Queensland**	
Rabies	https://www.qld.gov.au/health/condition/infections-and-parasites/viral-infections/rabies
**South Australia**	
Rabies virus and Australian bat lyssavirus - including symptoms, treatment and prevention	https://www.sahealth.sa.gov.au/wps/wcm/connect/public+content/sa+health+internet/conditions/infectious+diseases/rabies+virus+and+australian+bat+lyssavirus/rabies+virus+and+australian+bat+lyssavirus+-+including+symptoms+treatment+and+prevention
Rabies vaccines	https://www.sahealth.sa.gov.au/wps/wcm/connect/public+content/sa+health+internet/conditions/immunisation/vaccines/rabies+vaccines/rabies+vaccines
**Victoria**	
Rabies	https://www.betterhealth.vic.gov.au/rabies
**Western Australia**	
Rabies and Lyssaviruses	https://www.healthywa.wa.gov.au/Articles/N_R/Rabies-and-Lyssavirus
**U.S.A.**	
Rabies	https://wwwnc.cdc.gov/travel/diseases/rabies
Rabies	https://www.cdc.gov/rabies/index.html
What is Rabies?	https://www.cdc.gov/rabies/about.html

Animals and Rabies	https://www.cdc.gov/rabies/animals/index.html
Avoid risk of rabies from bats	https://www.cdc.gov/rabies/animals/bats/index.html
How is rabies transmitted?	https://www.cdc.gov/rabies/transmission/index.html
The Path of the Rabies Virus	https://www.cdc.gov/rabies/transmission/body.html
When should I seek medical attention?	https://www.cdc.gov/rabies/exposure/index.html
What kind of animal did you come in contact with?	https://www.cdc.gov/rabies/exposure/animals/index.html
Domestic Animals	https://www.cdc.gov/rabies/exposure/animals/domestic.html
Exotic Pet Species	https://www.cdc.gov/rabies/exposure/animals/exotic.html
Bats	https://www.cdc.gov/rabies/exposure/animals/bats.html
Other Wild Animals	https://www.cdc.gov/rabies/exposure/animals/other.html
Wildlife Reservoirs for Rabies	https://www.cdc.gov/rabies/exposure/animals/wildlife_reservoirs.html
What type of exposure occurred?	https://www.cdc.gov/rabies/exposure/type.html
Is the animal available for testing?	https://www.cdc.gov/rabies/exposure/testing.html
Rabies Prevention	https://www.cdc.gov/rabies/prevention/index.html
How can you prevent rabies in animals?	https://www.cdc.gov/rabies/prevention/animals.html
How can you prevent rabies in people?	https://www.cdc.gov/rabies/prevention/people.html
Rabies Postexposure Prophylaxis (PEP)	https://www.cdc.gov/rabies/medical_care/index.html
Human Rabies Immune Globulin	https://www.cdc.gov/rabies/medical_care/hrig.html
Rabies Vaccine	https://www.cdc.gov/rabies/medical_care/vaccine.html
Programs for Uninsured and Underinsured Patients	https://www.cdc.gov/rabies/medical_care/programs.html
Pre-exposure Prophylaxis (PrEP)	https://www.cdc.gov/rabies/prevention/pre-exposure_vaccinations.html

What are the signs and symptoms of rabies?	https://www.cdc.gov/rabies/symptoms/index.html
How is rabies diagnosed?	https://www.cdc.gov/rabies/diagnosis/index.html
Diagnosis in animals and humans	https://www.cdc.gov/rabies/diagnosis/animals-humans.html
Rabies in the U.S. and around the world	https://www.cdc.gov/rabies/location/index.html
Rabies in the U.S.	https://www.cdc.gov/rabies/location/usa/index.html
Cost of Rabies Prevention	https://www.cdc.gov/rabies/location/usa/cost.html
Is Rabies in Your State?	https://www.cdc.gov/rabies/location/usa/surveillance/index.html
Rabies around the World	https://www.cdc.gov/rabies/location/world/index.html
Rabies Status: Assessment by Country	https://www.cdc.gov/rabies/resources/countries-risk.html
Information for Travelers	https://www.cdc.gov/rabies/specific_groups/travelers/index.html
What if I receive treatment outside the United States?	https://www.cdc.gov/rabies/specific_groups/travelers/treatment_outside_us.html
About Rabies$^{\Delta}$	https://www.cdc.gov/rabies/about/index.html
Rabies Prevention and Control	https://www.cdc.gov/rabies/prevention/index.html
Rabies Status: Assessment by Country	https://www.cdc.gov/rabies/country-risk/index.html
When to Seek Medical Care for Rabies	https://www.cdc.gov/rabies/when-to-seek-care/index.html
Global Rabies: What You Should Know	https://www.cdc.gov/rabies/around-world/index.html
**CANADA**	
Rabies	https://travel.gc.ca/travelling/health-safety/diseases/rabies
Rabies: Symptoms and treatment	https://www.canada.ca/en/public-health/services/diseases/rabies.html
Prevention and risks	https://www.canada.ca/en/public-health/services/diseases/rabies/prevention.html
Monitoring	https://www.canada.ca/en/public-health/services/diseases/rabies/symptoms.html
Travel health advice	https://www.canada.ca/en/public-health/services/diseases/rabies/surveillance.html
**U.K.**	
Rabies	https://travelhealthpro.org.uk/factsheet/20/rabies

Rabies	https://www.fitfortravel.nhs.uk/advice/disease-prevention-advice/rabies
Overview	https://www.nhs.uk/conditions/rabies/
Vaccination	https://www.nhs.uk/conditions/rabies/vaccination/
Rabies information for travellers	https://www.gov.uk/government/publications/rabies-risks-for-travellers/rabies-information-for-travellers
Rabies information for travellers	https://assets.publishing.service.gov.uk/media/60a945dde90e071b5b4060f0/PHE_11543_Rabies_information_for_travellers_leaflet.pdf
**WHO**	
Rabies	https://www.who.int/news-room/fact-sheets/detail/rabies
Rabies overview	https://www.who.int/health-topics/rabies#tab=tab_1
Rabies symptoms	https://www.who.int/health-topics/rabies#tab=tab_2
Rabies treatment	https://www.who.int/health-topics/rabies#tab=tab_3
Frequently asked questions about rabies for the General Public	https://cdn.who.int/media/docs/default-source/ntds/rabies/rabies-general-public-faqs.pdf?sfvrsn=ac59055f_4

[Δ]Updated CDC website

Supplementary S2 – Search strategy

Figure S1 details the search strategy applied in this study. Search terms included "rabies"; "rabies information"; "rabies vaccination"; and "rabies and travel," coupled with the relevant country or jurisdiction. We selected Australia, Canada, New Zealand, the United States of America, and the United Kingdom as they have similar rabies profiles in which rabies is not endemic nor present in companion animals, hence similar messaging around rabies was expected. Only authorised websites, identified either through a government badge or official branding on the webpage, were assessed; thus, commercial sources of rabies information were excluded. We included websites that provided information on lyssaviruses if it was in conjunction with information on rabies, but excluded websites which were lyssavirus specific. Websites with a non-consumer audience, (such as those targeted towards healthcare providers and veterinarians), were also excluded.

We examined each webpage for "linked pages" which provided further information on rabies. Websites with a series of linked pages were considered as one resource. Each page of each website was saved as a PDF document to prevent the loss off resources in the event of a resource being updated by the administrator during the analysis period.

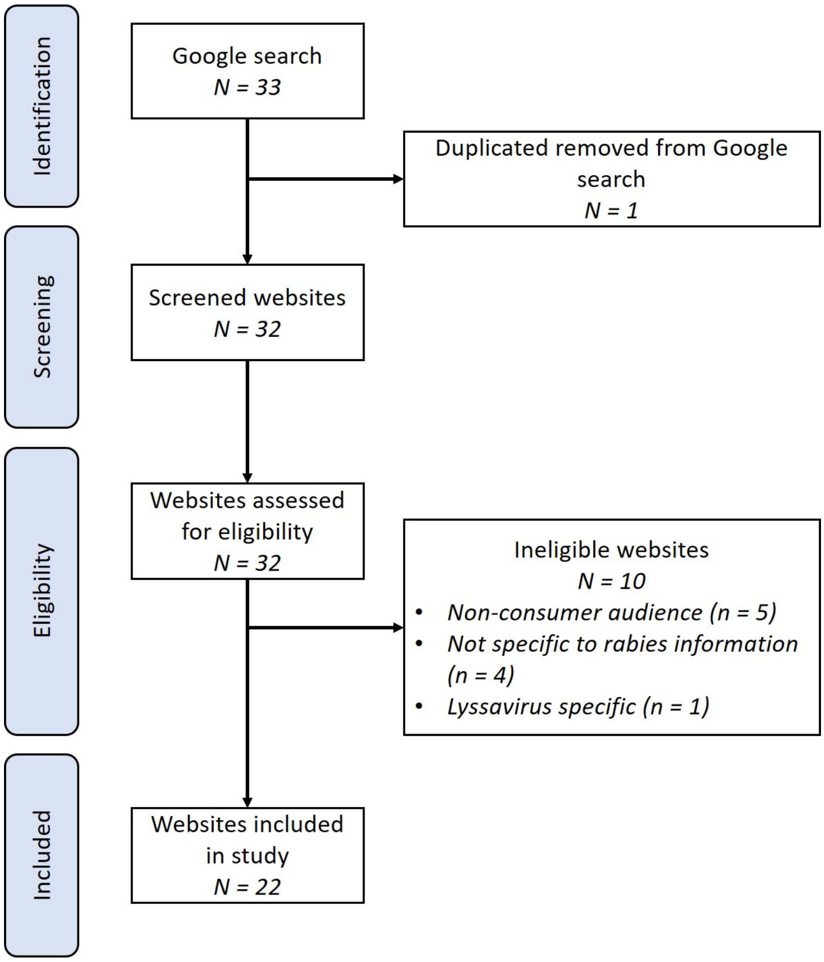

Figure S1. Website selection process

Supplementary S3 – Readability protocol

We prepared text following a protocol adapted from the guidelines developed by the SHeLL editor creators(1), and the methodology used by Mac et al.(2)

Full stops and colons that did not mark the end of a sentence (such as those within acronyms. mid-sentence, or as decimal points) were removed, as were headings of three words or less. Short sentences of three words of less were retained if not part of a bullet point.

URLs and short bullet points (three words or less) were excluded, while longer bullet points (four words or more) were retained. If the first bullet point was three words or less it was joined to the preceding sentence, with colons removed to avoid underestimating readability. Bullet points solely linking to other resources were excluded, but those with actionable content were included.

Figure captions and table contents were included. Table contents were converted into bullet points, with the same rules for bullet points applied. Roman numerals were changed to Arabic numerals.

Telephone numbers, footnotes, hyperlinked headings displayed as a table of contents or list at the beginning of sources, publication or update dates, reference lists, in-text references, and links to further information at the bottom of the page were removed.

Information not intended for consumers, whether implicitly or explicitly, was excluded from readability calculations, irrespective of whether it appeared on a separate page or alongside consumer-oriented content.

Supplementary S4 – Patient Education Materials Assessment Tool for printable materials (PEMAT-P) framework[‡]

Topic	Item	Description	Rating options
**Understandability**			
Content	1	The material makes its purpose completely evident.	Disagree = 0<br>Agree = 1
	2	The material does not include information or content that distracts from its purpose.	Disagree = 0<br>Agree = 1
Word choice and style	3	The material uses common, everyday language.	Disagree = 0<br>Agree = 1
	4	Medical terms are used only to familiarize audience with the terms. When used, medical terms are defined.	Disagree = 0<br>Agree = 1
	5	The material uses the active voice.	Disagree = 0<br>Agree = 1
Use of numbers	6	Numbers appearing in the material are clear and easy to understand.	Disagree = 0<br>Agree = 1<br>No use of numbers = N/A
	7	The material does not expect the user to perform calculations.	Disagree = 0<br>Agree = 1
Organisation	8	The material breaks or "chunks" information into short sections.	Disagree = 0<br>Agree = 1<br>Very short material = N/A
	9	The material's sections have informative headers.	Disagree = 0<br>Agree = 1<br>Very short material = N/A
	10	The material presents information in a logical sequence.	Disagree = 0<br>Agree = 1
	11	The material provides a summary.	Disagree = 0<br>Agree = 1<br>Very short material = N/A

Layout and design	12	The material uses visual cues (e.g., arrows, boxes, bullets, bold, larger font, highlighting) to draw attention to key points.	Disagree = 0<br>Agree = 1<br>Video = N/A
Use of visual aids	15	The material uses visual aids whenever they could make content more easily understood (e.g., illustration of healthy portion size).	Disagree = 0<br>Agree = 1
	16	The material's visual aids reinforce rather than distract from the content.	Disagree = 0<br>Agree = 1<br>No visual aids = N/A
	17	The material's visual aids have clear titles or captions.	Disagree = 0<br>Agree = 1<br>No visual aids = N/A
	18	The material uses illustrations and photographs that are clear and uncluttered.	Disagree = 0<br>Agree = 1<br>No visual aids = N/A
	19	The material uses simple tables with short and clear row and column headings.	Disagree = 0<br>Agree = 1<br>No tables = N/A
**Actionability**			
	20	The material clearly identifies at least one action the user can take.	Disagree = 0<br>Agree = 1
	21	The material addresses the user directly when describing actions.	Disagree = 0<br>Agree = 1
	22	The material breaks down any action into manageable, explicit steps.	Disagree = 0<br>Agree = 1
	23	The material provides a tangible tool (e.g., menu planners, checklists) whenever it could help the user take action.	Disagree = 0<br>Agree = 1
	24	The material provides simple instructions or examples of how to perform calculations.	Disagree = 0<br>Agree = 1<br>No calculations = N/A

	25	The material explains how to use the charts, graphs, tables, or diagrams to take actions.	Disagree = 0<br>Agree = 1<br>No charts / diagrams / tables / graphs = N/A
	26	The material uses visual aids whenever they could make it easier to act on the instructions.	Disagree = 0<br>Agree = 1

‡ Source: Shoemaker et al.(3) Full guide can be accessed at: https://www.ahrq.gov/health-literacy/patient-education/pemat.html

Supplementary S5 – Completeness framework

		**Scoring**		
**Item**	**Description**	**2 – complete**	**1 – partial**	**0 – not included**
**General rabies information**				
1	Introduction to rabies	Explains that rabies is caused by a virus AND That it only affects mammals OR Mentions a variety of species that it affects (e.g. dogs, bats, cats, humans)	Mentions that rabies is caused by a virus AND that it affects animals but does not provide further details of species affected	No introduction to rabies
2	Mode of transmission	Explains all modes of transmission (bites, scratches, licks to broken skin, mucous membranes)	Explains some but not all components of transmission	No information on transmission
3	Geographic distribution of rabies	Indicates that there are differences in the presence of rabies and the affected species in different countries AND Provides a destination-based indication of rabies distribution and/or risk globally or domestically, such as a map or table of endemic countries or areas OR Includes an embedded link to a resource that provides this (e.g. https://apps.who.int/neglected_diseases/ntddata/rabies/rabies.html). Rate as "partial" if a link is only included at the bottom of the page or under a "further information" heading	Mentions the presence of endemic areas without detailed geographic or species distribution. E.g. "Rabies is a risk in many parts of the world, with most cases occurring in Africa and Asia". (gives general continents or regions) OR States that rabies may occur in wildlife, without providing further geographic information	No information on geographic distribution
4	Individual risk factors	Describes a range of specific factors that increase exposure risk (such as age, outdoor activities, travel duration, working with or handling animals) or	Includes a general statement such as "risks may vary according to individual factors," or "your doctor can	Does not mention any specific risk factors

		that limit access to post-exposure prophylaxis (e.g. travel to remote areas with limited healthcare access)	do a risk assessment" but does not specify characteristics that may influence risk.	
5	Prevention – non-pharmacological	Provides specific advice on non-pharmacological measures to reduce exposure risk, such as not feeding, patting or playing with animals.	Includes a general statement, such as "avoid contact with animals" but does not elaborate.	Does not mention non-pharmacological prevention methods.
6	Prevention – PrEP	Provides specific information on pre-exposure prophylaxis including who should consider or get vaccinated and the implications of receiving PrEP , including that it simplifies post-exposure prophylaxis in the event of a bite by eliminating the need for rabies immune globulin and reducing the number of rabies vaccine doses required after an exposure	Provides a general statement around PrEP but does not provide details (e.g. "Rabies can be prevented with vaccination – speak to your doctor if travelling to a country where there is a rabies virus risk) and/or does not explain the implications of receiving PrEP	Does not mention PrEP
7	Post-exposure – immediate action	Provides information on immediate actions following a potential rabies exposure including both (1) wash the wound immediately and thoroughly with soap and water and (2) seek medical attention as soon as possible	Includes a general statement about seeking medical care and/or wound care without specifically mentioning when and how	Does not give the reader any information on what to do if exposed
8	Post-exposure – rabies immune globulin	Provides information on the need for rabies immune globulin as soon as possible after an exposure in previously unvaccinated persons<br>AND<br>Explains what RIG is and/or why it is necessary (e.g. RIG is an injectable product containing antibodies that give immediate protection against rabies until	Includes a general statement on the need for rabies immune globulin as soon as possible but no further details. (i.e. does not mention what RIG is or why RIG is important)	Does not mention post-exposure rabies immune globulin (RIG)

		the body can respond to the vaccine and make antibodies of its own).		
9	Post-exposure – rabies vaccination	Provides information on the need for rabies vaccination as soon as possible after an exposure. Includes specific information on the number of doses required in different situations (e.g. previously vaccinated vs. unvaccinated)<br>AND<br>Some indication of the schedule or timeframe (e.g. those who have not been previously vaccinated will need 4-5 doses over a period of 14-28 days)	Mentions that PEP is required as soon as possible after an exposure, but does not go into specifics (i.e. does not detail number of doses, schedule, approach to previously vaccinated vs. unvaccinated individuals)	Does not mention PEP
10	Availability of treatment for rabies	States that there is no specific treatment available for rabies and that once symptoms arise it is almost always fatal, but that the disease can be prevented by seeking post-exposure prophylaxis (RIG and vaccine)<br>AND<br>Gives a destination-based availability index of RIG (including availability in home country) and/or vaccine or links to a resource that does (either globally or domestically).	States that there is no specific treatment available for rabies and that once symptoms arise it is almost always fatal<br>AND<br>Provides a general statement that PEP or RIG may not be available in all countries or destinations but no further details (i.e. locations mentioned are not specific / does not mention availability in home country)	Does not mention that treatment is unavailable<br>OR<br>Does not mention "treatment" at all<br>OR<br>Does not give destination-based information on the availability of RIG or PEP
11	Risk of disease following an exposure	States that the risk of disease can vary according to the person's vaccination history and immune status and at least 1 other of the following criteria:<br>. animal species<br>· the type, extent, and location of the exposure	Mentions that the risk following an exposure varies but does not give specific examples of what may alter a person's risk	Does not mention that risk of disease post-exposure may vary.

12	Diagnosis	Includes information on testing of humans and animals. States that there are no tests to diagnose rabies before the onset of symptoms in humans. Includes something around testing in animals, e.g. that it should be assumed that any animal in an endemic area has rabies unless testing proves otherwise (e.g. if public health officials catch and test the animal)	Only includes information on testing of humans or animals.<br>OR<br>States only that there are no tests to diagnose rabies before the onset of symptoms	Does not mention diagnosis at all
13	Clinical presentation	States that rabies is nearly always fatal once symptoms appear<br>AND<br>Includes a description of a range of signs/symptoms (e.g. anxiety, confusion, agitation, delirium, abnormal behaviour, hallucinations, hydrophobia (fear of water), and insomnia)	States that rabies is nearly always fatal but lacks detail regarding symptoms	Does not mention any of the symptoms related to rabies infection<br>AND<br>does not mention that rabies is nearly always fatal once symptoms appear
14	Natural course of the disease	Indicates that the incubation period is variable and provides an indication of the range (e.g. usually between 3 and 12 weeks and/or ranges from 1 week to a few years). Explains that symptoms progress over a period of days to weeks and end in death.	Provides an indication of the progress of disease but not a specific timeframe (e.g. first there is an incubation stage, then prodromal, then acute disease, then death)	Does not state the disease timeline
**Information specific to vaccines (pre- and post-exposure)**				
15	Vaccine efficacy	Provides some numerical indication of the proportion of people who develop an appropriate immune response following rabies vaccination.. Indicates that the role of PrEP is to prime the immune	States that the rabies vaccine is effective at preventing rabies but does not provide a numerical indication<br>OR	Does not detail the efficacy nor necessity of PrEP

		response and simplify post-exposure treatment by removing the need for RIG and reducing the number of vaccine doses requires.	Does not explain the specific implications of receiving PrEP.	
16	Duration of protection	Indicates the duration of protection of a primary course of PrEP either by stating the time period of protection or the timing of booster doses (e.g. after 2 doses, protection last up to 3 years and a booster dose is required for protection beyond this OR after 3 doses, routine booster doses are not recommended except in x circumstances)	Provides a general statement on the duration of protection but does not provide specific details. May say something like "a booster dose may be required" but gives no numerical schedule/timeline.	Does not state any timeframe for protection duration
17	Number and schedule of pre-exposure doses required	Provides details on the number of doses required to confer protection (2 or 3 doses acceptable)<br>AND<br>States spacing e.g. for 3 doses (days 0, 7 and between 21 to 28) or 2 doses (days 0 and 7)	States that multiple doses will are required for full protection, but does not give a numerical value<br>OR<br>Does not give information on spacing of doses	Does not state how many doses are required<br>OR<br>Does not state any timeframe for PrEP
18	Availability of different schedules	Describes the availability of different schedules and modes of administration (e.g. mentions 2- versus 3-dose options and/or intramuscular vs. intradermal)<br>AND<br>Explains the rationale for selecting one over the other (e.g. intradermal to reduce costs for a family or group)	Mentions that there are different schedules and modes of administration but does not provide further details.	Does not mention that there are different schedules nor that there are different modes of administration available
19	Cost of vaccination	Explains that there is an out-of-pocket cost associated with receiving PrEP and provides a numerical indication of the cost of vaccination (either per dose or for a full course).	Mentions that there are costs associated with PrEP and/or PEP but does not provide a numerical value	Does not mention the cost of the vaccine or that there is an associated cost

		AND<br>Indicates that there may also be costs associated with PEP for travellers.		
20	Vaccine side effects/adverse events	Provides a description of a range of potential vaccine side effects such as a sore arm, pain, and redness at the site of injection, fever, malaise, headaches, dizziness, nausea, and a raised itchy rash.<br>AND<br>Offers insight into the relative frequency of side effects, highlighting which are more or less common, and includes information on how long typically last.	Offers a general statement about side effects, such as describing them as mild and possibly including pain at the injection site. Only mentions a few side effects without providing specific details on their frequency or duration.	Does not mention or state any potential vaccine side effects
21	Vaccine safety based on individual factors	Indicates that rabies vaccines are safe.<br>AND<br>Includes information on 2 or more of the following individual factors: age, allergies, pregnant women, immunosuppressed. E.g. mentions that people with an egg allergy should only receive specific brands (Merieux or Verorab, NOT Rabipur)<br>OR<br>Includes information on vaccination for special groups such as pregnant women and immunosuppressed individuals (e.g. may mention that vaccines can be given to immunosuppressed individuals but immune responses may be blunted.)	Includes a general statement such as "rabies vaccines are safe" but does not provide specific information for special groups. Acknowledges that there may be contraindications and to speak to a healthcare provider for more information	Does not mention any contraindications or that there may be contraindications for specific groups
22	Type of vaccine	Provides a description of the types of rabies vaccines available (at a minimum should indicate that these are inactivated	States that there are different vaccines available but does	Does not state the type of vaccine

		or non-live, can also include the names of the different vaccines available)<br>AND<br>Explains that they are interchangeable	not provide any specific details of each.	
23	Additional dose requirement for those previously vaccinated	Provides specific details of when and for whom booster doses are indicated (Must mention that people that have been previously vaccinated must have additional doses in the event of an exposure)	States that a booster dose may be required but does not elaborate on when or why.	Does not mention booster doses

Supplementary S6 – Country-specific rabies status information

**Website**	**Tool Name/type**	**Description**	**Audience**	**URL**
World Health Organisation	Rabies data base	Colour-coded map which describes the presence of dog-mediate rabies globally.	Consumer	https://www.who.int/data/gho/data/themes/topics/rabies
Centers for Disease Control	Rabies Status: Assessment by country	Users select a country and are provided with information on whether rabies or lyssaviruses are present, the availability of RIG and PEP, and whether the county has robust surveillance and control programs	Consumer	https://www.cdc.gov/rabies/country-risk/index.html

References

1. Ayre J, Bonner C, Muscat DM, Dunn AG, Harrison E, Dalmazzo J, et al. Multiple automated health literacy assessments of written health information: development of the SHeLL (Sydney Health Literacy Lab) health literacy editor v1. JMIR Form Res. 2023;7:e40645.
2. Mac OA, Muscat DM, Ayre J, Patel P, McCaffery KJ. The readability of official public health information on COVID-19. Med J Aust. 2021;215(8):373-5.
3. Shoemaker SJ, Wolf MS, Brach C. Development of the Patient Education Materials Assessment Tool (PEMAT): a new measure of understandability and actionability for print and audiovisual patient information. Patient Educ Couns. 2014;96(3):395-403.
